# Right ventricular involvement in Tako-tsubo cardiomyopathy - insights from cardiovascular magnetic resonance

**DOI:** 10.1186/1532-429X-17-S1-P280

**Published:** 2015-02-03

**Authors:** Caroline Scally, Christopher J Neil, Janaki Srinivasan, Baljit Jagpal, Bernice K Ng, Michael P Frenneaux, John Horowitz, Dana K Dawson

**Affiliations:** Cardiology, University of Aberdeen, Aberdeen, UK

## Background

It has been recently suggested that patients with Tako-tsubo cardiomyopathy (TTC) who exhibit right ventricular (RV) involvement at Echocardiography may have a worse prognosis. The aim of the current study was to prospectively evaluate the extent of RV involvement acutely and at follow up using gold-standard cardiac magnetic resonance (CMR).

## Methods

21 patients, mean age 66 (range 41-87 years) with a clear diagnosis of TTC (14 with ST-elevation, 16 with apical ballooning ) and emotional trigger were prospectively studied. CMR-derived LV and RV volumes and EF, RV shapes, RV wall motion index (WMSI, 6-segment model) and Echocardiography derived Pulmonary artery pressure (Pap), tricuspid annular E',A',S', pansystolic excursion (TAPSE) were measured acutely (day 0-3) and after 4 months follow-up.

## Results

Eleven patients demonstrated RV involvement on CMR - in contrast, RV wall motion abnormalities were identified in only 6 patients on Echocardiography Patients were grouped according to the "acute" RV-WMSI on CMR: WMSI=1 (Group A, n=10) and WMSI>1 (Group B, n=11).

*In the acute phase*, LVEF was significantly lower in Group B compared with Group A (48±10% vs 62±6%, p=0.02), but this did not reach statistical significance for RVEF (58±13% vs 65±7%, p=ns). However, Pap was significantly higher acutely in Group B compared to Group A (40±15 mmHg vs 28±6 mmHg, p=0.04). There were no significant differences between Groups for RV volumes (raw and indexed), E', A', S' or TAPSE.

*At follow-up*, LVEF improved significantly in both groups (62±6% to 66±6% in Group A, p= 0.01 and 48±10% to 63±6% in Group B, p<0.01). Pap decreased significantly in Group B (from 40±15 to 28±8, p=0.018). RV-WMSI normalized in all but one in Group B.

## Conclusions

CMR detected RV involvement in 52% of patients presenting with acute TTC vs only 29% detected on Echocardiography and should be used as a gold-standard. WMSI and Pap are the best markers to identify these patients.

## Funding

Grant:G13/10 from Tenovus Scotland.

